# Romiplostim N01 accelerates platelet engraftment in autologousstem cell transplantation using non-cryopreserved peripheral blood stem cells for plasma cell neoplasms

**DOI:** 10.3389/fimmu.2026.1813060

**Published:** 2026-04-21

**Authors:** Xianfu Sheng, Qijia Zheng, Jingjing Xiang, Nanxi Dong, Yuechao Zhao, Huijin Hu, Lili Qian, Wenbin Liu, Jianping Shen, Baodong Ye, Yu Zhang

**Affiliations:** 1 Department of Hematology, The First Affiliated Hospital of Zhejiang Chinese Medical University (Zhejiang Provincial Hospital of Chinese Medicine), Hangzhou, China; 2 The First Medical College of Zhejiang Chinese Medical University, Hangzhou, China; 3 Department of Laboratory, The First Affiliated Hospital of Zhejiang Chinese Medical University (Zhejiang Provincial Hospital of Chinese Medicine), Hangzhou, China

**Keywords:** autologous hematopoietic stem cell transplantation, multiple myeloma, non-cryopreserved stem cells, romiplostim N01, systemic light-chai amyloidosis, thrombopoietin receptor agonist

## Abstract

**Background:**

Delayed platelet engraftment remains a major limitation of autologous stem cell transplantation (ASCT) for plasma-cell neoplasms. Romiplostim N01, a thrombopoietin receptor agonist, may enhance early megakaryocytic recovery, while the use of non-cryopreserved peripheral blood stem cells (PBSCs) eliminates dimethyl-sulfoxide–related toxicity and reduces procedural cost.

**Methods:**

This retrospective study evaluated 15 patients receiving non-cryopreserved PBSCs and early Romiplostim N01 after ASCT and compared them with 21 historical controls who received cryopreserved PBSCs and recombinant human thrombopoietin. We tried to compare time to engraftment, transfusion burden, hospitalization duration and cost, safety, hematologic responses and survival outcomes.

**Results:**

Platelet engraftment occurred significantly earlier in the Romiplostim N01 cohort (median 11 vs. 13 days; *P* = 0.008), and complete platelet recovery by day +30 was higher (100% vs. 66.7%; *P* = 0.027). Neutrophil recovery, transfusion requirements, and hospitalization duration were comparable between groups. Total hospitalization cost was markedly lower with Romiplostim N01 (77, 609 ± 21, 624 vs. 106, 188 ± 14, 910 CNY; *P* < 0.001). The two patient groups also demonstrated comparable safety profiles, treatment responses, and survival outcomes.

**Conclusions:**

Romiplostim N01 safely accelerates thrombopoietic recovery and substantially reduces cost when combined with non-cryopreserved PBSCs. This strategy represents a practical and economically favorable supportive-care model for ASCT.

## 1 Introduction

Autologous stem cell transplantation (ASCT) remains a cornerstone of treatment for transplant-eligible multiple myeloma (MM) and systemic light-chain (AL) amyloidosis (1–3). High-dose melphalan (HD-Mel) conditioning reliably deepens hematologic responses; however, it often induces prolonged cytopenias (4–6). Among these, delayed platelet engraftment is particularly consequential, increasing the risk of hemorrhage, prolonging transfusion dependence, necessitating extended hospitalization, and elevating overall healthcare cost (7). Despite its clinical relevance, strategies optimized specifically to enhance thrombopoietic recovery after ASCT remain limited.

Current supportive measures rely primarily on platelet transfusion and, in select centers, short-term administration of recombinant human thrombopoietin (rhTPO) (8, 9). Platelet transfusion is effective for acute bleeding prevention but imposes significant logistic and cost burdens, and repetitive exposure increases the risk of alloimmunization and transfusion reactions (8). rhTPO, while theoretically attractive, has a short half-life, variable efficacy in the post-HD-Mel setting, and risks related to antibody formation (10–12). These limitations underscore the need for more effective, sustained, and biologically targeted thrombopoietic support.

Thrombopoietin receptor agonists (TPO-RAs) directly stimulate the c-Mpl receptor, activating downstream signaling pathways—including JAK-STAT, PI3K-AKT, and MAPK—that drive megakaryocyte proliferation, endomitosis, and proplatelet formation (13–15). TPO-RAs do not generate neutralizing antibodies, making them widely used for thrombocytopenia after hematopoietic stem cell transplantation (HSCT) (14, 16–20). Recently, several studies have investigated the prophylactic use of TPO-RAs (e.g., romiplostim, eltrombopag, hetrombopag) to enhance platelet engraftment in HSCT, with promising results (10, 21–24), but the optimal timing, dosing, and combination strategies remain to be defined. Romiplostim N01, a domestically manufactured biosimilar of the original romiplostim molecule (13), offers theoretical advantages in mitigating HD-Mel–related marrow suppression. Yet evidence supporting its use in ASCT remains limited.

In parallel, renewed clinical interest has emerged regarding non-cryopreserved peripheral blood stem cells (PBSCs) (25, 26). Cryopreservation requires controlled-rate freezing, dimethyl-sulfoxide (DMSO) handling, and long-term liquid-nitrogen storage; DMSO infusion itself carries risks of nausea, hypertension, bradycardia, and rare neurotoxicity (27). Non-cryopreserved PBSCs, when infused within short intervals after collection, can provide hematopoietically competent grafts while reducing toxicity, shortening processing time, and significantly lowering cost (28, 29).

Integrating early Romiplostim N01 with non-cryopreserved PBSCs represents a biologically and operationally synergistic strategy: pharmacologic acceleration of megakaryopoiesis coupled with simplified graft workflow. However, this combined approach has not been formally evaluated. Therefore, we conducted a retrospective cohort study to evaluate the efficacy and safety between patients receiving Romiplostim N01 plus non-cryopreserved PBSCs and historical controls receiving cryopreserved PBSCs and rhTPO.

## 2 Patients and methods

### 2.1 Study design and setting

This single-center retrospective cohort study included adult patients diagnosed with multiple MM or AL amyloidosis who underwent their first ASCT at the First Affiliated Hospital of Zhejiang Chinese Medical University. In the observation group, we collected data from consecutive patients who received non-cryopreserved PBSCs and prophylactic Romiplostim N01, between May 2024 and July 2025. Meanwhile, patients who underwent ASCT from January 2022 to December 2024 were included as historical controls, selecting those receiving cryopreserved PBSCs with rhTPO as supportive therapy. This study was approved by the Institutional Ethics Committee of the First Affiliated Hospital of Zhejiang Chinese Medical University (Approval No. 2025-KLS-945-01) and complied with the principles of the Declaration of Helsinki. Given the retrospective design and the use of anonymized clinical data only, the ethics committee approved a waiver of informed consent, as the study posed minimal risk and re-contacting all participants was not feasible.

### 2.2 Patient eligibility

Patients were eligible if they were ≥18 years old, had confirmed MM or AL amyloidosis, and were deemed appropriate candidates for ASCT based on institutional criteria. Adequate hepatic, renal, and cardiopulmonary function was required. Patients were excluded for uncontrolled active infection, pregnancy, a history of thromboembolic events within the preceding 3 months, prior exposure to TPO-RAs (defined as any use of a TPO-RA before ASCT), or incomplete medical records. Baseline clinical characteristics—including disease subtype, remission status, cytogenetic or FISH risk, induction regimen, comorbidity profile, and organ involvement—were abstracted from electronic medical documentation.

### 2.3 Mobilization, apheresis, and graft handling

Stem-cell mobilization for the Romiplostim N01 cohort consisted of granulocyte colony-stimulating factor (G-CSF) 10 μg/kg/day for 5–6 days. Plerixafor (20 mg) was administered if peripheral CD34^+^ counts were <20/μL on the anticipated day of apheresis. Apheresis was performed using continuous-flow cell separators, with the goal of collecting ≥2.0 × 10^6^ CD34^+^ cells/kg. Mobilized PBSCs were stored at 4 °C and reinfused within 24–48 hours without cryopreservation.

Mobilization regimens in the historical cohort included G-CSF alone or cyclophosphamide plus G-CSF, depending on practices during the corresponding period. PBSCs were processed using standard DMSO cryopreservation. Products were frozen in controlled-rate freezers and stored in vapor-phase liquid nitrogen until infusion. Cryopreserved graft infusion involved standard premedication protocols to mitigate DMSO-related reactions.

### 2.4 Conditioning regimen and supportive care

All patients received HD-Mel, administered at 200 mg/m² for eligible patients or 140 mg/m² for individuals with renal impairment or advanced age, per institutional guidelines. Stem-cell infusion occurred on day 0. Supportive care included antibacterial, antifungal, and antiviral prophylaxis; antiemetics; tumor lysis prevention when clinically indicated; oral cryotherapy; and aggressive intravenous hydration. G-CSF was restarted on day +5 and continued until neutrophil recovery. Red-cell and platelet transfusions followed institutional thresholds and were administered based on clinical status rather than prespecified numeric triggers.

### 2.5 Romiplostim N01 and rhTPO administration

Romiplostim N01 was initiated on day +1 at a weekly dose of 5 μg/kg. Dose escalation to 10 μg/kg was permitted if platelet engraftment (≥20 × 10^9^/L) had not occurred by day +14, and treatment continued until platelet counts surpassed 75 × 10^9^/L or a maximum of four doses was given. In the historical cohort, rhTPO (300 U/kg/day) was administered subcutaneously when platelet counts dropped below 75 × 10^9^/L and was continued for up to 14 days if needed. No other TPO-RAs were used during the study.

### 2.6 Definitions and outcome measures

The primary endpoint was time to platelet engraftment, defined as the first of three consecutive days with platelet counts ≥20 × 10^9^/L without transfusion for at least seven days. Secondary endpoints included time to neutrophil engraftment (ANC ≥0.5 × 10^9^/L for three consecutive days), complete platelet recovery (≥100 × 10^9^/L by day +30), duration of severe thrombocytopenia (<20 × 10^9^/L), transfusion burden (single-donor platelet units and packed red cells), hospital length of stay, hospitalization cost, adverse events, hematologic responses and survival outcomes.

Response assessments followed International Myeloma Working Group criteria and Mayo criteria (30, 31), evaluated at baseline, post-induction, and day +90 post-ASCT. Progression-free survival (PFS) and overall survival (OS) were calculated from the date of ASCT to the time of disease progression, relapse, death, or last follow-up. Cumulative incidence of relapse was estimated with non-relapse mortality as a competing risk.

### 2.7 Statistical analysis

Continuous variables were summarized as median (range) and compared using the Mann–Whitney U test. Categorical variables were compared using Fisher’s exact test. Time-to-event endpoints were analyzed using Kaplan–Meier curves, compared using the log-rank test. Competing-risk analyses used Gray’s test. A two-sided *P* < 0.05 was considered statistically significant. Statistical analyses were performed using SPSS (version 26.0) and R (version 4.2.2).

## 3 Results

### 3.1 Patient characteristics

A total of 15 patients underwent non-cryopreserved PBSC ASCT with Romiplostim N01 support (Romiplostim N01 group), while 21 historical controls received cryopreserved PBSC ASCT with rhTPO (Control group). Baseline demographic and disease-related variables—including age, sex, disease subtype, International Staging System (ISS) or Mayo stage, disease status prior to transplantation, hematopoietic cell transplant comorbidity index (HCT-CI) score, plerixafor use and single day apheresis—were generally comparable between the two groups (**Table 1**).

**Table 1:** Characteristics of plasma cell neoplasms patients and grafts.

Variable	Romiplostim N01 group (N = 15)	Control group (N = 21)	*P* value
Median age, years (range)	59.8 (50-79)	61 (45-78)	0.849
Sex, no. (%)			
Male Female	11 (73.3) 4 (26.7)	13 (61.9) 8 (38.1)	0.721
Diagnosis, no. (%)			
MM AL amyloidosis	12 (80) 3 (20)	20 (95.2) 1 (4.8)	0.287
ISS stage of MM, no. (%)	N=12	N=20	
I II III	4 (33.3) 4 (33.3) 4 (33.3)	7 (35) 6 (30) 7 (35)	1.000
Mayo 2012 stage of AL amyloidosis, no. (%)	N=3	N=1	
I II III IV	0 1 (33.3) 2 (66.7) 0	0 0 0 1 (100)	0.500
Median time from diagnosis to ASCT, months, (range)	5.5 (3-9)	7 (5-32)	0.002
Median number of induction cycles (range)	4 (2-6)	5 (4-16)	0.024
Disease status before ASCT, no. (%)			
SD PR VGPR CR	1 (6.7) 3 (20) 7 (46.7) 4 (26.7)	1 (4.8) 6 (28.6) 9 (42.9) 5 (23.8)	0.954
HCT-CI score, no. (%)			
0 1-2 3	9 (60) 5 (33.3) 1 (6.7)	11 (52.4) 9 (42.9) 1 (4.8)	0.838
Plerixafor use, no. (%)	9 (60)	7 (33.3)	0.175
Single day apheresis, no. (%)	5 (33.3)	4 (19)	0.443
Dose of Melphalan, no. (%)			
200mg/m^2^ 140mg/m^2^	9 (60) 6 (40)	14 (66.7) 7 (33.3)	0.736
Stem cells CD34^+^, ×10^6^/kg, median (range)	4.53 (1.26-10.05)	5.60 (1.24-22.04)	0.499

MM, Multiple myeloma; AL, Immunoglobulin light-chain; ISS, International Staging System; ASCT, Autologous hematopoietic stem cell transplantation; SD, Stable disease; PR, Partial response; VGPR, Very good partial response; CR, Complete response; HCT-CI, hematopoietic cell transplantcomorbidity index; n, Number.

Patients in the Romiplostim N01 cohort tended to proceed to transplantation earlier in their disease course, with a shorter median interval from diagnosis to ASCT (5.5 vs. 7.0 months; *P* = 0.002) and fewer induction cycles prior to transplantation (median 4 vs. 5 cycles; *P* = 0.024) (**Table 1**), reflecting earlier disease control and transplantation timing rather than differences in disease biology. The CD34^+^ cell dose infused was similar between the cohorts, measuring 4.53 ×10^6^/kg (range 1.26–10.05) in the Romiplostim N01 group and 5.60 ×10^6^/kg (range 1.24–22.04) in controls (*P* = 0.499) (**Table 1**), confirming comparable graft quality. Conditioning intensity and melphalan dosing strategies were identical between groups, ensuring consistent transplant-related toxicity exposure (**Table 1**).

### 3.2 Hematopoietic engraftment

**Figure 1:**
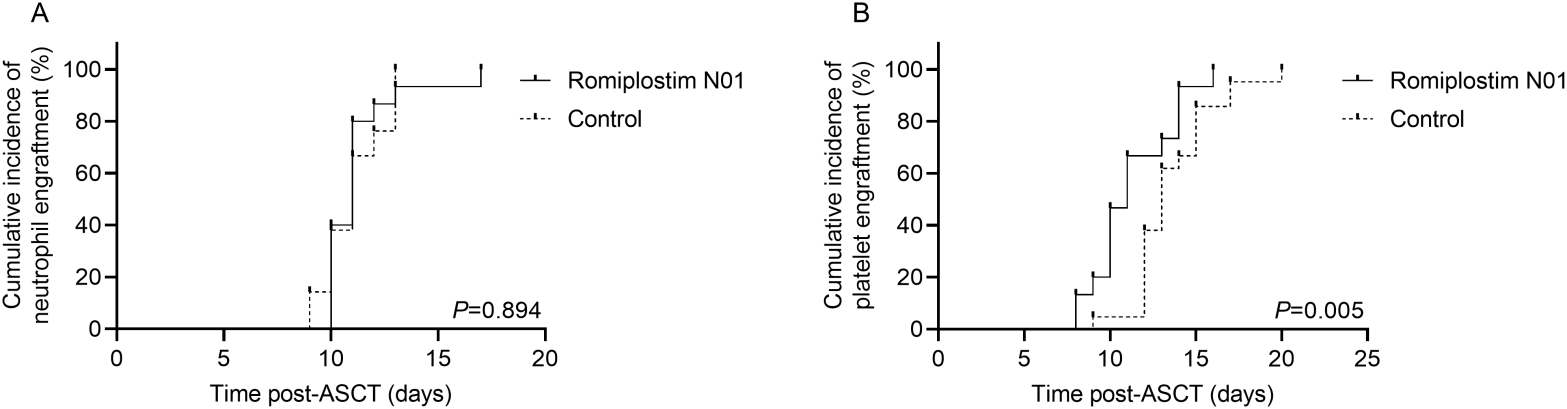
Cumulative incidence of neutrophil and platelet engraftment. Time-to-event analysis was used to calculate the cumulative incidence of neutrophil **(A)** and platelet **(B)** engraftment with death in aplasia as competing risks. ASCT: Autologous hematopoietic stem cell transplantation;*P* < 0.05 was considered significant.

All patients in both cohorts achieved hematopoietic engraftment. By day +30, Median time to neutrophil engraftment were similar between groups (11 days [range 10–17]) vs. controls (11 days [range 9–13]; *P* = 0.899) (**Table 2**). The neutrophil engraftment kinetics were also similar (**Figure 1A**), indicating equivalent early myeloid reconstitution. In contrast, platelet engraftment occurred significantly earlier in the Romiplostim N01 group, with a median of 11 days (range 8–16) compared with 13 days (range, 9–20) in controls (*P* = 0.008) (**Table 2**). This advantage was consistent across individual patients and was reflected in a left-shifted cumulative recovery curve (**Figure 1B**). Moreover, the quality of platelet reconstitution differed substantially. By day +30, all patients in the Romiplostim N01 cohort (100%) achieved complete platelet recovery (≥100 ×10^9^/L), whereas only 66.7% of controls reached this threshold (*P* = 0.027) (**Table 2**). No patient in the Romiplostim N01 cohort experienced a secondary platelet decrease within 90 days post-transplant. This divergence in sustained thrombopoietic improvement highlights the biological impact of Romiplostim N01 on megakaryocyte maturation beyond mere early engraftment.

**Table 2:** Comparisons of engraftment, transfusion burden, and hospitalization metrics post-ASCT.

Variable	Romiplostim N01 group (N = 15)	Control group (N = 21)	*P* value
Median neutrophil engraftment, days (range)	11 (10-17)	11 (9-13)	0.899
Median platelet engraftment, days (range)	11 (8-16)	13 (9-20)	0.008
Graft failure, no. (%)	0	0	
Platelet counts ≥ 100 × 10^9^/L, no. (%)	15 (100)	14 (66.7)	0.027
Median duration of severe thrombocytopenia (platelet counts < 20 × 10^9^/L), days (range)	5 (0-8)	4 (0-14)	0.800
Platelet transfusion requirements, no. (%)	14 (93.3)	18 (85.7)	0.626
Number of SDP transfusion, (range)	1 (0-3)	1 (0-3)	0.924
Volume of transfused platelet, units	22 (0-56)	18 (0-59)	0.704
RBC transfusion requirements, no. (%)	2 (13.3)	0	0.167
Length of hospital stay, days (range)	30 (22-49)	32 (25-45)	0.693
Total cost of ASCT (CNY) – mean ± SD	77609 ± 21624	106188 ± 14910	<0.001

ASCT, Autologous hematopoietic stem cell transplantation; SDP, Single donor platelet; RBC, Red blood cell; CNY, Chinese Yuan;n: Number.

Despite these improvements, the duration of severe thrombocytopenia (<20 ×10^9^/L) was similar between cohorts (median 5 days [0–8] vs. 4 days [0–14]; *P* = 0.800) (**Table 2**), suggesting that Romiplostim N01 primarily accelerated recovery after nadir rather than modifying nadir depth.

### 3.3 Transfusion burden and hospitalization outcomes

Platelet transfusion requirements were comparable between groups. 93.3% of patients receiving Romiplostim N01 and 85.7% of controls required at least one platelet transfusion (*P* = 0.626). The number of single-donor platelet (SDP) units administered per patient (median 1 unit [0–3] vs. 1 unit [0–3]) and the total units of platelets transfused (22 units [0–56] vs. 18 units [0–59]) did not differ significantly (*P* = 0.924 and *P* = 0.704, respectively) (**Table 2**). These findings were consistent with the similar depth and duration of severe thrombocytopenia observed in each group. Red-blood-cell transfusion was required in two patients in the Romiplostim N01 cohort and none in the control cohort, a difference not statistically significant (*P* = 0.167) (**Table 2**).

The median length of hospitalization was comparable, measuring 30 days (range 22–49) in the Romiplostim N01 cohort and 32 days (25–45) in controls (*P* = 0.693). However, total hospitalization cost showed a marked and clinically meaningful reduction in the Romiplostim N01 group. The mean total cost was 77, 609 ± 21, 624 CNY compared with 106, 188 ± 14, 910 CNY in the control cohort (*P* < 0.001) (**Table 2**), reflecting a nearly 27% reduction. The largest contributions to cost reduction were elimination of cryopreservation-related processing expenses and reduced reliance on rhTPO, despite the added cost of Romiplostim N01. Collectively, these findings underscore the economic efficiency of combining Romiplostim N01 with non-cryopreserved PBSCs.

### 3.4 Safety

Romiplostim N01 demonstrated a favorable safety profile, with no unexpected adverse events observed. The frequency and grade of common non-hematologic toxicities—including mucositis, gastrointestinal symptoms (nausea, vomiting, and diarrhea), and febrile neutropenia—were similar across cohorts and consistent with those typically encountered following HD-Mel ASCT (**Table 3**).

**Table 3:** Comparisons of safety profiles.

Variable	Romiplostim N01 group (N = 15)	Control group (N = 21)	*P* value
Gastrointestinal adverse events			
Mucositis, no. (%)	9 (60)	15 (71.4)	0.499
Nausea/vomiting, no. (%)	15 (100)	20 (95.2)	1.000
Diarrhea, no. (%)	11 (73.3)	20 (95.2)	0.138
Infectious adverse events			
Febrile neutropenia, no. (%)	15 (100)	20 (95.2)	1.000
Bacteremia, no. (%)	1 (8.3)	5 (23.8)	0.379
Other adverse events			
Hepatic injury, no. (%)	2 (12.5)	7 (33.3)	0.248
Cardiovascular events	0	0	
Severe infusion-related reactions	0	0	
Thromboembolic events	0	0	
ICU admission	0	0	

ICU, Intensive care unit; n, Number.

Bacteremia occurred in 8.3% of patients receiving Romiplostim N01 and 23.8% of controls (*P* = 0.379), and elevations in hepatic transaminases occurred in 12.5% vs. 33.3% (*P* = 0.248) (**Table 3**), respectively. Although numerically lower in the Romiplostim N01 group, these differences did not reach statistical significance. No thromboembolic events, severe infusion-related reactions, acute cardiovascular instability, or ICU admissions were reported in either group. Importantly, no cases of symptomatic marrow fibrosis or romiplostim-associated hypersensitivity reactions were observed. Together, these findings support the safety and tolerability of early Romiplostim N01 administration following ASCT.

### 3.5 Post-transplant responses and survival

**Figure 2:**
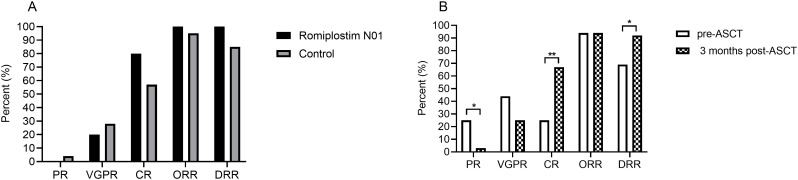
Hematologic responses to ASCT. Comparisons of efficacy endpoints (PR, VGPR, CR, ORR, and DRR) are shown between the Romiplostim N01 group and the control group in **(A)**, whereas the comparisons of the same endpoints before and after ASCT (at 3 months) among all patients are presented in **(B)**. ASCT: Autologous hematopoietic stem cell transplantation; PR: Partial response; VGPR: Very good partial response; CR: Complete response; ORR:Overall response rate; DRR: Deep response rate. *P* < 0.05 was considered significant.

Firstly, at the 3-month post-transplant efficacy assessment, no significant differences were observed between the Romiplostim N01 and control groups in terms of partial response (PR) rate, very good partial response (VGPR) rate, complete response (CR) rate, overall response rate (ORR, CR + VGPR + PR), or deep response rate (DRR, CR + VGPR) (**Figure 2A**), indicating that Romiplostim N01 did not impair disease control. To evaluate transplantation’s impact on disease control, we pooled patients from both the Romiplostim N01 and control groups and compared pre- versus post-transplant responses. The CR rate increased from 25% pre-ASCT to 67.7% post-ASCT (*P* < 0.001), resulting in significantly higher DRR post-ASCT (91.7% vs. 69.4%, *P* = 0.035), consistent with the deepened response from HD-Mel conditioning. However, ORR failed to improve (94.4% vs. 94.4%, *P* = 1.000) as non-remission rates remained unchanged (**Figure 2B**).

**Figure 3:**
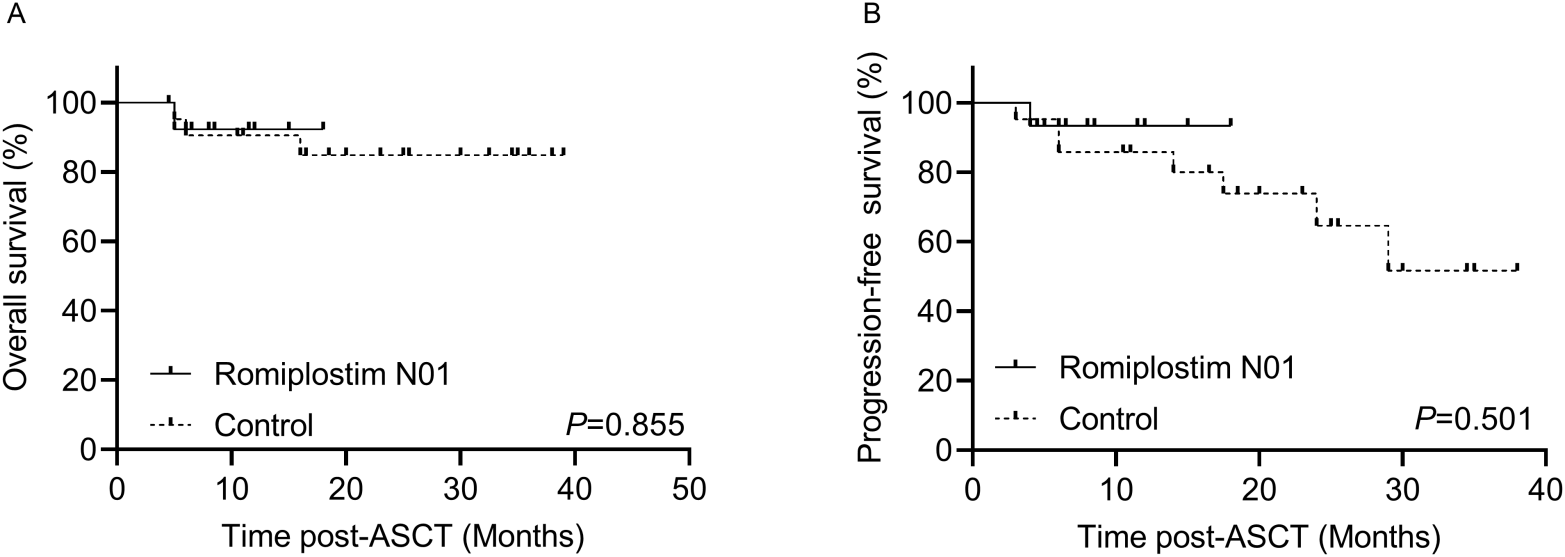
Survival outcomes. **(A)** The comparison of estimated 2-year OS between Romiplostim N01 and control groups (92.3% ± 7.4% vs. 84.8% ± 8.1%, *P* = 0.855). **(B)** The comparison of estimated 2-year PFS between Romiplostim N01 and control groups (93.3% ± 6.4% vs. 51.7% ± 15.2%, *P* = 0.501). ASCT, Autologous hematopoietic stem cell transplantation. *P* < 0.05 was considered significant.

**Figure 4:**
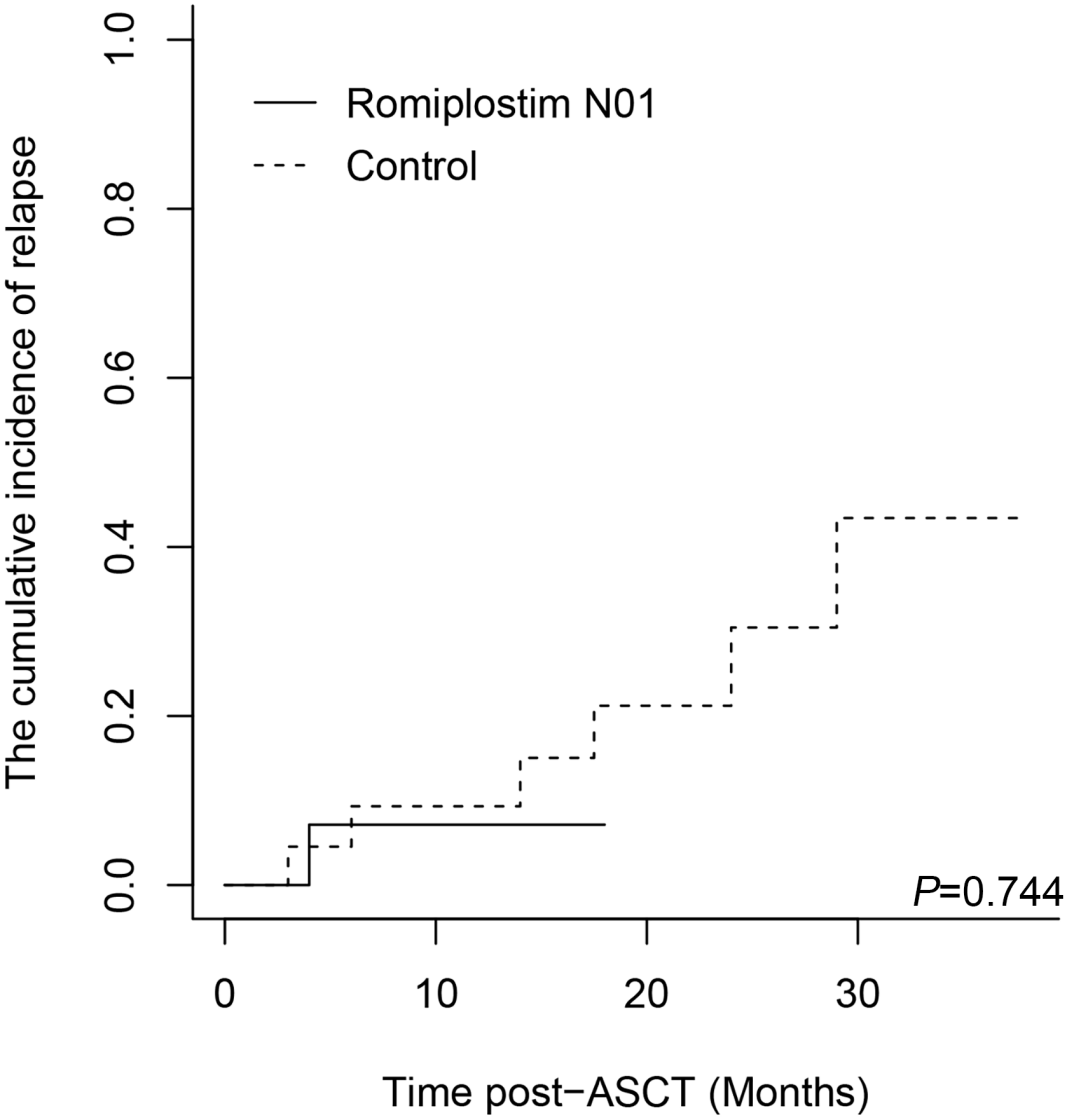
Cumulative incidence of relapse, with death as competing risk. Comparison of cumulative relapse rates between the Romiplostim N01 group and the control group. ASCT, Autologous hematopoietic stem cell transplantation. *P* < 0.05 was considered significant.

With a median follow-up of 13.5 months, early survival outcomes were comparable between groups. Estimated 2-year OS was 92.3% ± 7.4% in the Romiplostim N01 group and 84.8% ± 8.1% in the control cohort (*P* = 0.855) (**Figure 3A**). Estimated 2-year PFS was 93.3% ± 6.4% vs. 51.7% ± 15.2% (*P* = 0.501) (**Figure 3B**), with the wider confidence interval in controls reflecting greater heterogeneity and fewer events. The cumulative incidence of relapse with death as a competing risk did not differ significantly (7.14% ± 0.51% vs. 43.42% ± 2.74%; *P* = 0.744) (**Figure 4**). No transplant-related mortality within the first 100 days occurred in either cohort.

## 4 Discussion

This retrospective cohort study demonstrates that early administration of Romiplostim N01 following ASCT, combined with the use of non-cryopreserved PBSCs, leads to significantly accelerated platelet engraftment, more consistent complete platelet recovery, and substantially reduced hospitalization cost, while maintaining an excellent safety profile. Our evaluation of Romiplostim—a TPO-RA more accessible than rhTPO across many regions—provides findings that are more generalizable to diverse real-world transplant settings. These findings collectively highlight the feasibility, clinical value, and economic advantages of integrating a TPO-RA–based thrombopoietic support strategy into routine ASCT practice for plasma-cell neoplasms.

Recent studies present conflicting evidence regarding the ability of non-cryopreserved PBSCs to shorten engraftment time (28, 32, 33). A Worldwide Network for Blood & Marrow Transplantation meta-analysis of 1, 686 patients confirmed the feasibility and safety of non-cryopreserved PBSCs transplantation. This analysis reported a median platelet engraftment time of 15.3 days, aligning with the 15-day median reported by Jacinth et al. in *Blood* (25, 34). In contrast, our study demonstrated a significantly shorter median platelet engraftment time of 11 days. Given comparable median CD34^+^ cell counts across the studies, the accelerated platelet recovery observed in our cohort may be attributable to the administration of Romiplostim N01.

The two-day improvement in platelet engraftment observed in the Romiplostim N01 cohort is modest but clinically meaningful and aligns with the known pharmacologic activity of TPO-RAs (7). HD-Mel induces profound megakaryocytic suppression by impairing stromal integrity, damaging vascular niches, and temporally arresting megakaryocyte endomitosis (35, 36). Endogenous TPO levels, though increased during cytopenia, remain insufficient to overcome this transient marrow injury. Romiplostim N01, through c-Mpl receptor binding and activation of JAK-STAT, PI3K-AKT, and MAPK pathways, provides a supraphysiologic stimulus that restores megakaryopoiesis during this vulnerable post-conditioning window (13). The improvement in both the speed and quality of thrombopoietic recovery—evidenced by universally achieved complete platelet recovery by day +30—is consistent with this mechanistic rationale and suggests a coordinated enhancement of megakaryocyte maturation and proplatelet formation.

These findings expand the existing body of evidence for TPO-RAs in transplantation. Previous studies evaluating TPO-RAs in HSCT have largely focused on salvage therapy for poor graft function or delayed engraftment, often requiring prolonged administration over several weeks (14, 16–20). Recent studies have explored the prophylactic administration of TPO-RAs in ASCT with the aim of enhancing platelet recovery (10, 21, 22). Scordo et al. reported that weekly romiplostim (starting 3 μg/kg on day +1) did not accelerate platelet engraftment or reduce transfusions versus historical controls, though platelet counts at days +21/+30 were higher (21). Prakash et al. reported that single doses of romiplostim (250 μg on day +3) and pegylated G-CSF accelerated engraftment (9.72 vs. 12.57 days) and reduced transfusion needs (22). Xu et al. showed that hetrombopag plus rhTPO accelerated platelet recovery (9 vs. 14 days) and reduced transfusions (10). Taken together, these studies have limitations but collectively suggest that TPO-axis stimulation is feasible and may offer clinical benefit in this setting. In our study, the median platelet engraftment time was 11 days—longer than in Prakash and Xu but 2 days shorter than our historical controls—and we uniquely achieved 100% complete platelet recovery by day +30, accompanied by a 27% reduction in hospitalization cost. Contrary to prior reports, our study demonstrates that combining Romiplostim N01 with non-cryopreserved PBSCs accelerates engraftment, enhances recovery quality, and offers significant cost savings—outcomes not described previously. The economic advantage observed in the Romiplostim N01 cohort is another compelling aspect of this strategy. Hospitalization cost was reduced by nearly 27%, driven primarily by elimination of cryopreservation-related expenses, including DMSO handling, controlled-rate freezing, liquid-nitrogen storage, cryoprotective equipment, infusion-related monitoring, and management of DMSO-associated adverse events (25, 28, 29). The use of non-cryopreserved PBSCs avoids these steps entirely and simplifies the logistical framework of ASCT processing. Notably, this cost reduction was achieved despite the addition of Romiplostim N01. A cost comparison shows that Romiplostim N01 (∼1, 500 CNY/dose; median 2–3 doses, total 3, 000–4, 500 CNY) replaced rhTPO (∼1, 000 CNY/dose; median 12 doses, total ∼12, 000 CNY). Thus, the shorter treatment duration with Romiplostim N01 yields substantial pharmacologic savings, contributing alongside cryopreservation elimination to the overall economic benefit.

The safety profile of Romiplostim N01 observed in this study is consistent with prior experience in immune thrombocytopenia and chemotherapy-induced thrombocytopenia (16, 37–39). No thromboembolic complications, infusion reactions, exacerbations of mucositis or gastrointestinal events, hepatic toxicity, or marrow fibrosis was observed. The absence of cardiovascular or pro-thrombotic signals is especially reassuring, given concern for thrombopoietic overstimulation in the peri-engraftment period. Importantly, there was no evidence that Romiplostim N01 adversely affected disease control; depth of hematologic response increased appropriately after ASCT, and early PFS and OS outcomes were comparable to those in the control cohort.

The clinical implications of these findings are noteworthy. In many transplant centers—particularly those with high patient volume or limited cryopreservation infrastructure—simplifying the PBSCs workflow could significantly reduce resource strain (27). Non-cryopreserved PBSCs infusion eliminates DMSO-associated toxicities, supports more predictable scheduling, and reduces dependence on cryopreservation facilities (34). When paired with Romiplostim N01, this approach provides a coherent and rational framework for reducing the medical and financial burden of ASCT without structurally altering conditioning strategies or transfusion practices.

Nevertheless, this study has limitations. Its retrospective nature introduces inherent risks of selection bias, although baseline characteristics were comparable between cohorts. The modest sample size limits the power to detect rare adverse events, small differences in survival outcomes, or subgroup-specific effects (e.g., MM vs. AL). Follow-up duration remains relatively short, precluding assessment of late relapses, long-term marrow stability, or delayed bone marrow fibrosis. Additionally, although the control group reflected contemporaneous institutional practice, evolving supportive-care protocols may have introduced unmeasured confounders. Future larger prospective studies comparing the efficacy and safety of non-cryopreserved PBSCs ASCT with or without Romiplostim N01 should be conducted to confirm these findings. Such studies should also evaluate optimal dosing strategies, long-term safety outcomes, and the cost-effectiveness of this approach in diverse healthcare settings.

Despite these limitations, the strengths of the study are notable: uniform conditioning regimens, consistent definitions of engraftment and toxicity, stable institutional transplant practices across the study period, and integration of both clinical and economic endpoints. The internal consistency across engraftment acceleration, improved platelet recovery quality, and substantial cost reduction strongly supports the biological and operational validity of this combined strategy.

## 5 Conclusion

This study provides hypothesis-generating real-world evidence that early administration of Romiplostim N01 accelerates platelet recovery, enhances the quality of thrombopoiesis, and significantly reduces hospitalization cost without compromising safety when used in combination with non-cryopreserved PBSCs during ASCT for plasma-cell neoplasms. These findings support the integration of Romiplostim N01 into routine thrombopoietic support for ASCT, particularly in centers seeking to streamline graft processing and reduce resource utilization.

## Ackowledgments

The authors would like to extend their sincere gratitude to Professor Jindan Yu for her language polishing of this paper.

## Data Availability

The raw data supporting the conclusions of this article will be made available by the authors, without undue reservation.
